# Value of contrast-enhanced ultrasonography of the carotid artery for evaluating disease activity in Takayasu arteritis

**DOI:** 10.1186/s13075-019-1813-2

**Published:** 2019-01-16

**Authors:** Ling-Ying Ma, Chao-Lun Li, Li-Li Ma, Xiao-Meng Cui, Xiao-Min Dai, Ying Sun, Hui-Yong Chen, Bei-Jian Huang, Lin-Di Jiang

**Affiliations:** 1 Department of Rheumatology, Zhongshan Hospital, Fudan University, 180 Fenglin Road, Shanghai, 200032 People’s Republic of China; 2 Department of Ultrasound, Zhongshan Hospital, Fudan University, Shanghai, People’s Republic of China; 3 Center of Clinical Epidemiology and Evidence-based Medicine, Fudan University, Shanghai, People’s Republic of China

**Keywords:** Takayasu arteritis, Contrast-enhanced ultrasonography, Carotid artery

## Abstract

**Aims:**

To assess the value of contrast-enhanced ultrasonography (CEUS) for monitoring disease activity of Takayasu arteritis (TA).

**Methods:**

TA patients were recruited in a Chinese TA clinical center from January 2016 to September 2017. The physician global assessment was used as the referential standard for disease activity. Clinical data, acute phase reactants, and CEUS scans were simultaneously recorded at baseline and after a 3-month therapy.

**Results:**

A total of 84 TA patients were enrolled, and 47 (55.95%) cases were active at baseline. Macaroni sign and entire artery involvement were characteristic findings of CEUS in TA. The average vascular full thickness of the carotid artery in active TA patients was significantly higher than that in inactive patients (2.36 ± 0.86 vs. 1.79 ± 0.49 mm; *p* = 0.001). Severe neovascularization (grade 2) was observed in 29 active cases (61.70%) and in 9 inactive cases (24.32%) (*p* = 0.001). Receiver operating characteristic analysis showed that the combination of CEUS parameters (cutoff of thickness was 1.75 mm or neovascularization grade 2) and erythrocyte sedimentation rate (ESR) (cutoff of 20 mm/H) could help differentiate between active and inactive TA patients with a sensitivity and specificity of 81.1% and 81.5%, respectively. Youdon’s index was 0.626. Furthermore, our study found that patients with decreased ESR and C-reactive protein (CRP) still had a progression of vascular wall inflammation at 3 months of follow-up.

**Conclusions:**

The evaluation of vascular inflammation by CEUS is more sensitive than acute phase reactants. Neovascularization can still be observed in the vascular lesion sites of those who have reached clinical remission after treatment. Thus, CEUS can be used as an alternative method to assess disease activity for TA patients.

## Introduction

Takayasu arteritis (TA) is a type of chronic inflammatory vasculitis that develops mainly in Asian women of childbearing age. The condition primarily involves the aorta and its branches, as well as the ascending aorta, abdominal aorta, renal artery, and pulmonary artery. Pathological data suggest that inflammation progresses from the adventitia to the intima and eventually encompasses the whole layer, leading to stenosis, occlusion, or expansion and consequently to organ ischemia and infarction. Once TA is diagnosed, comprehensive disease assessments, including disease activity, vascular stenosis, organ function, and drug-related adverse events, should be conducted to guide the therapeutic strategy. However, up to date, the biggest challenge is the lack of quantitative, reliable, and effective measures to monitor disease activity in TA.

Commonly used parameters, such as erythrocyte sedimentation rate (ESR) and C-reactive protein (CRP), lack specificity and may not reflect the local inflammation of the vascular wall. Imaging techniques play an important role in the diagnosis and surveillance of TA. Previous studies have shown that color Doppler ultrasonography (CDUS) and computed tomography angiography (CTA) can be used for diagnosing TA, although these methods are not significantly associated with disease activity. Magnetic resonance angiography (MRA) yields unclear results when assessing the carotid artery, possibly because of considerable interference. Positron emission tomography/computed tomography (PET/CT) cannot be widely used for monitoring disease activity because of the high cost and risk of nuclear radiation [1, 2]. Hence, it is vital to identify novel imaging methods for monitoring disease activity.

Contrast-enhanced ultrasonography (CEUS) has been widely used in atherosclerotic disease as the supplementary imaging technique for CDUS. The use of ultrasound contrast agents facilitates the identification and quantification of intraplane neovascularization in the carotid artery. Retrospective studies indicated that the enhancement of neovascularization in carotid plaques on CEUS is associated with clinical symptoms, cardiovascular events, and plaque types [3–5]. Some recent studies indicated CEUS as a potential imaging marker of disease activity in large-vessel vasculitis, which may be more sensitive than the laboratory indicators [6–8].

According to the Numano classification [9], TA can be classified into five types. Although the frequency of each type differs between races and regions, the involvement of carotid arteries is common. Imaging findings in our TA cohort showed that type I and type V were the most common, and carotid artery involvement was commonly seen. Moreover, previous studies have shown that approximately 45 to 84% of patients with TA have carotid involvements [10]; hence, CEUS has shown its advantage on evaluating vascular inflammation of large-vessel vasculitis. In the present study, we aimed to assess the value of CEUS for monitoring disease activity in patients with TA.

## Patients and methods

### Patient population

We enrolled 84 patients diagnosed with TA (based on the 1990 American College of Rheumatology diagnostic criteria [11]) in the TA clinical center of Zhongshan Hospital Fudan University from January 2016 to September 2017. The exclusion criteria were as follows: (1) TA patients without carotid artery involvement; (2) those with contraindications for the use of ultrasound contrast agents, such as acute cardiac failure, unstable angina, known right-to-left shunts, and acute endocarditis; and (3) those with allergy against ultrasound contrast agents. A total of 37 atherosclerosis patients were also included as controls in the study, and these patients underwent only CDUS. The study was approved by the ethics committee of Zhongshan Hospital Fudan University. Informed written consent was obtained from each patient.

### Data collection

Demographic parameters, clinical symptoms, and physical examination findings of TA patients were electronically recorded. Serological tests, such as assessments for complete blood count, ESR, CRP, serum amyloid A (SAA), immunoglobulin, and cytokines, were performed within 3 days before CEUS examination. Physician’s global assessment (PGA) [12] was used as the gold standard for disease activity assessment, which comprised newly issued or recently aggravated neck pain, dizziness, pulseless or other ischemic symptoms, elevated ESR, and new lesions on imaging examination. The criteria defined by Kerr et al. [13] and the Indian Takayasu Clinical Activity Score (ITAS2010) [12] were also evaluated. All patients were followed up after 3 months.

### CDUS examination

All patients in both the TA group and the atherosclerosis control group accepted carotid CDUS, which aimed to identify the specific characteristics of TA. CDUS was performed on a Philips Elite ultrasound instrument (Philips Medical Systems, Bothell, WA, USA), with a L9-3 linear array probe. The extracranial carotid arteries (common carotid artery, internal carotid artery, and external carotid artery) were assessed by the same experienced physician. In the supine position, all patients turned their heads laterally and fully exposed their necks. For each patient, the bilateral carotid arteries were examined and the wall thickness of the normal carotid artery bifurcation was measured as a standard image of intima-media thickness (IMT). The diameter of the lesion wall was also measured as the wall thickness. The mechanical index was 0.07, and gain was 70%. The instrument parameters were kept consistent for all patients.

### CEUS examination

CEUS was performed at the thickest site of the common carotid artery found by CDUS in TA patients. The US contrast agent (2 mL; SonoVue, Bracco, Italy) was injected through the middle elbow vein and immediately followed by 5.0 mL of saline. The instrument’s built-in timer was initiated simultaneously to prepare for image acquisition. The probe was fixed for 2 min, and images were continuously and automatically acquired; dynamic images were then stored in the instrument hard disk. A specific medical digital image transmission format (digital imaging and communication in medicine; DICOM) was used to store images for offline analysis.

**Fig. 1:**
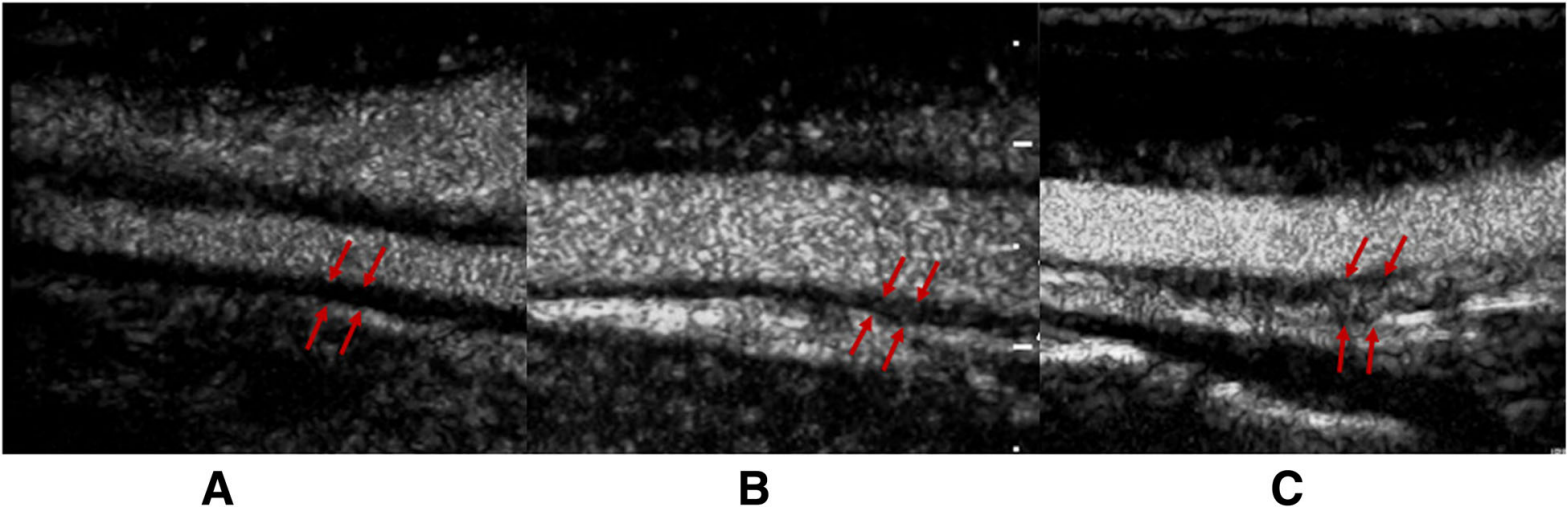
The neovascularization degree of the thickened wall on CEUS. **a** Grade 0, no vascularization. **b** Grade 1, limited or moderate vascularization. **c** Grade 2, severe vascularization

The degree of neovascularization at the thickening wall on CEUS was defined as follows [8]: grade 0, no vascularization, representing no moving microbubbles in the thickened carotid lesions; grade 1, limited or moderate vascularization, representing limited or moderate visible appearance of moving microbubbles in the thickened carotid lesions; and grade 2, severe vascularization, representing extensive wall vascularization with a clear visible appearance of moving microbubbles (Fig. 1).

### Statistical analysis

The cohort of the TA clinical center was prospectively studied. According to the optimal cutoff value for PGA concentration, TA patients were divided into an active and inactive group. Categorical variables were compared between the two groups using the *χ*^2^ test. All continuous variables were described as mean ± SD and compared using Student’s *t* test. The clinical value of the vascularization grade of carotid lesions as a disease activity parameter was evaluated using the logistic regression model. Odds ratios (ORs) and 95% confidence intervals (CIs) were examined. Sensitivity and specificity of a selection of the following screening measures were tested using the receiver operating characteristic (ROC) analysis for individual and combined tests: CEUS parameters, ESR, and CRP. Significance was defined at *p* < 0.05. Statistical analysis was performed using SPSS for Windows (version 22).

## Results

### Patient characteristics

Both newly treated patients and those undergoing long-term treatment were included in the study. The average patient age was 31.88 ± 10.01 years (range, 14–57 years). The female-to-male ratio was 6.64:1. The disease duration ranged from 1 month to 27 years (median, 29 months). The longest follow-up duration was 1.5 years (range, 0–1.5 years; median, 3 months). Imaging tests were done in all the patients including CTA (4 cases), vascular ultrasound (20 cases), or MRA (78 cases). According to the 1996 Numano classification, types I, IIa, IIb, and V were observed in 44% (*n* = 37), 9.5% (*n* = 8), 2.4% (*n* = 2), and 44% (*n* = 37) cases in the study, respectively. At baseline, 49 patients had received treatment previously. All of these patients received glucocorticoid (initial dosage, 0.8 mg/kg) combined with immunosuppressors, including 5 azathioprine (50 mg/day), 7 methotrexate (10–15 mg/week), 5 cyclophosphamide (15 mg/kg/month), 15 mycophenolate mofetil (30 mg/kg/day), 16 leflunomide (20 mg/day), and 2 tocilizumab (8 mg/kg/month). The other 35 patients had not received any treatments previously.

On the basis of PGA, 47 patients were active and the other 37 were inactive. Forty-one active (87.23%) patients complained of new symptoms such as neck pain, dizziness, pulseless, fatigue, fever, or asymmetrical blood pressure. Furthermore, only one inactive patient complained of dizziness. In the present study, 20 (23.81%) patients had comorbidities including hypertension, whereas 7 (8.33%) had cerebral infarction.

The average disease duration in the active group was shorter than that in the inactive group (34.96 ± 5.10 vs 84.34 ± 13.87 months, *p* = 0.001). With regard to serological tests, there was a significant difference in ESR, CRP, SAA, and platelet count between the two groups. Compared with the inactive group, both Kerr scores (2.40 ± 0.92 vs 1.24 ± 0.83, *p* < 0.001) and ITAS2010 index (4.68 ± 4.93 vs 1.84 ± 3.98, *p* = 0.004) were higher in the active group (Table 1).

**Table 1:** Characteristics of TA patients

	Total	Active	Inactive	*p*
*N*	84	47	37	
Sex, female (%)	73 (86.90)	40 (85.11)	33 (89.19)	0.415
Age, years	31.88 ± 10.01	29.64 ± 8.78	34.73 ± 10.86	0.023
Disease duration, months	47.49 ± 66.03	34.96 ± 5.10	84.34 ± 13.87	0.001
Clinical manifestations				
Hypertension (%)	20 (23.81)	7 (14.89)	13 (35.14)	0.029
Asymmetrical blood pressure (%)	3 (3.57)	3 (6.38)	0	0.170
Pulseless (%)	6 (7.14)	6 (12.77)	0	0.032
Fatigue (%)	7 (8.33)	7 (14.89)	0	0.016
Dizziness (%)	9 (10.71)	8 (17.02)	1 (2.70)	0.035
Neck pain (%)	14 (16.67)	14 (29.79)	0	< 0.001
Fever (%)	3 (3.57)	3 (6.38)	0	0.170
Received therapies (%)	49 (58.33)	20 (42.55)	29 (78.38)	0.001
Laboratory examination				
ESR, mm/H	37.46 ± 32.95	50.72 ± 34.55	20.53 ± 21.25	< 0.001
CRP, mg/L	27.13 ± 44.19	41.37 ± 53.85	8.53 ± 11.66	0.001
SAA, mg/L	99.71 ± 176.38	144.01 ± 212.72	32.29 ± 52.87	0.017
Platelet count, ×10^9^/L	301.23 ± 110.81	327.85 ± 123.58	267.22 ± 81.64	0.013
Kerr scores	1.89 ± 1.05	2.40 ± 0.92	1.24 ± 0.83	< 0.001
ITAS 2010 scores	3.43 ± 4.73	4.68 ± 4.93	1.84 ± 3.98	0.004
Ultrasound index				
Carotid artery wall thickness, mm	2.12 ± 0.77	2.36 ± 0.86	1.79 ± 0.49	0.001
Carotid artery diameter, mm	3.73 ± 2.19	3.91 ± 1.92	3.48 ± 2.51	0.424
Diffuse lesions (%)	72 (85.71)	40 (85.11)	32 (86.49)	0.557
Proportions of vascular stenosis (%)	59 (70.24)	29 (61.70)	30 (81.08)	0.044
Proportions of vascular occlusion (%)	10 (11.90)	2 (4.26)	8 (21.62)	0.019
Carotid artery peak flow rate, m/s	1.37 ± 0.99	1.59 ± 1.04	1.08 ± 0.85	0.019
Carotid RI	0.73 ± 0.13	0.73 ± 0.13	0.72 ± 0.13	0.915
CEUS carotid wall vascularization grade 2 (%)	38 (45.24)	29 (61.70)	9 (24.32)	0.001

*TA* Takayasu arteritis, *ESR* erythrocyte sedimentation rate, *CRP* C-reactive protein, *SAA* serum amyloid A, *RI* resistance index, *CEUS* contrast-enhanced ultrasonography

### Comparisons between TA and atherosclerosis patients at baseline

In comparison with atherosclerosis patients, TA patients were much younger (31.88 ± 10.01 vs 62.68 ± 8.78 years, *p* < 0.001), and most (86.90%) were female. On CDUS, local vascular lesions were more common in cases with carotid atherosclerosis (33 [89.19%] vs 12 [14.29%], *p* < 0.001). Carotid thickening in TA mainly occurred in the initial segment, followed by the entire carotid artery. On contrast imaging of TA, carotid plaques were found to be more common at the bifurcation of the carotid artery, and the echo of the plaques was not uniform. A significantly narrower lumen (3.73 ± 2.19 vs 6.49 ± 0.84 mm, *p* < 0.001) and thicker artery wall (2.12 ± 0.78 vs 1.33 ± 0.94 mm, *p* < 0.001) were observed in cases with TA. The macaroni sign was a characteristic manifestation of TA and was not found in any atherosclerosis patient (Table 2).

**Table 2:** The difference between TA and atherosclerosis patients in CDUS

	TA	Carotid atherosclerosis	*p*
*N*	84	37	
Age, years	31.88 ± 10.01	62.68 ± 8.78	< 0.001
Sex, female (%)	73 (86.90)	6 (16.22)	< 0.001
Unilateral carotid artery lesion (%)	17 (20.20)	10 (27.03)	0.479
Local vascular lesion (%)	12 (14.29)	33 (89.19)	< 0.001
Initial lesion of carotid artery (%)	81 (96.40)	3 (8.11)	< 0.001
Bifurcation lesion of carotid artery (%)	2 (2.38)	33 (89.19)	< 0.001
Hypoechoic lesions (%)	84 (100)	6 (16.22)	< 0.001
Carotid artery diameter, mm	3.73 ± 2.19	6.49 ± 0.84	< 0.001
Carotid artery wall thickness, mm	2.12 ± 0.78	1.33 ± 0.94	< 0.001
Macaroni sign (%)	84 (100)	0	< 0.001
Carotid artery peak flow rate, m/s	1.37 ± 0.99	1.21 ± 1.07	0.466
Carotid RI	0.73 ± 0.13	0.74 ± 0.07	0.565

*TA* Takayasu arteritis, *CDUS* color Doppler ultrasonography, *RI* resistance index

### Comparison of CDUS/CEUS parameters between the active and inactive TA groups

The average vascular full thickness of the carotid artery was greater in the active group (2.36 ± 0.86 vs 1.79 ± 0.49 mm, *p* = 0.001) than that in the inactive group. We observed a wider range of diffuse lesions in the active group, although the difference was not significant. Both the wall thickness and diffuse lesions indicate the presence of vascular damage. Furthermore, in the inactive group, the proportion of luminal stenosis and luminal occlusion was higher (*p* < 0.05; Table 1).

Carotid CEUS showed severe vascularization (grade 2) in 29 (61.70%) active cases and 9 (24.32%) inactive cases (*p* = 0.001), which indicated that vascular inflammation could be observed in clinical inactive patients (Table 1).

### Relationship between CEUS parameters and disease activity biomarkers

**Fig. 2:**
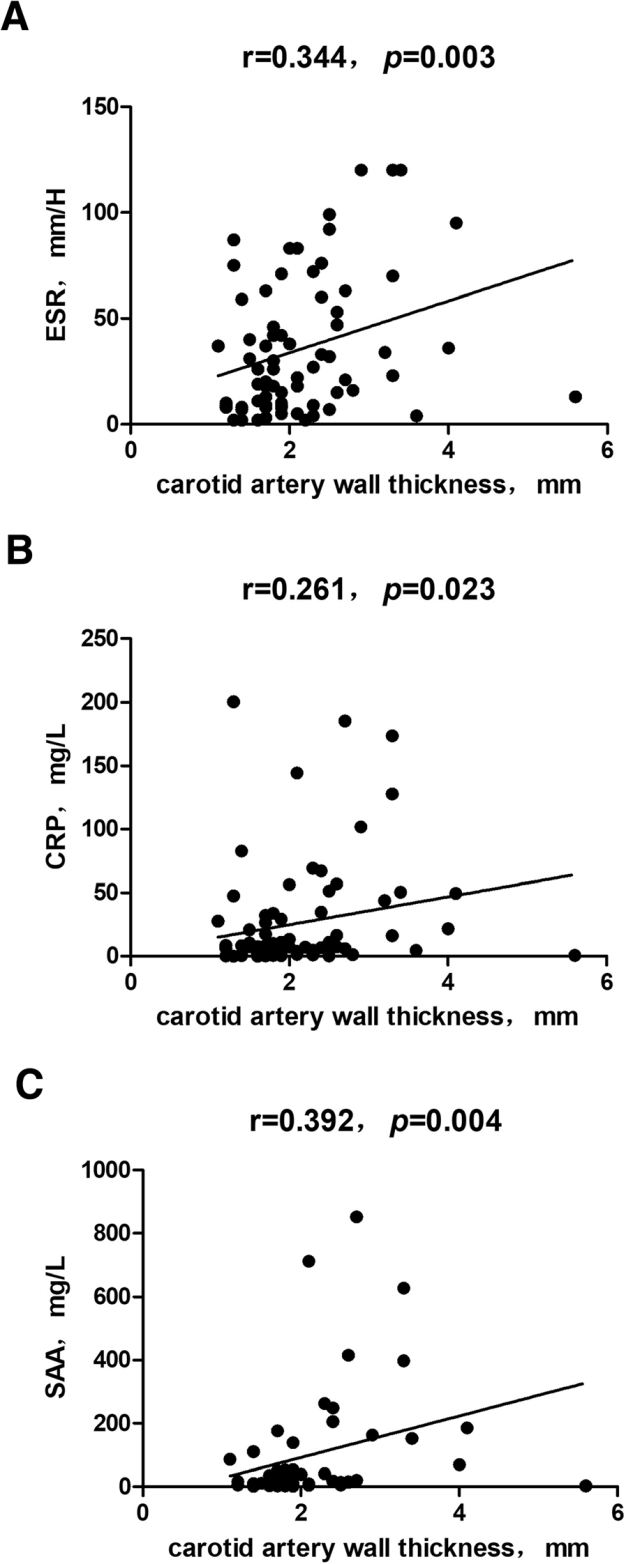
The relationship between carotid artery wall thickness and disease activity markers. **a** The relationship between carotid artery wall thickness and ESR. **b** The relationship between carotid artery wall thickness and CRP. **c** The relationship between carotid artery wall thickness and SAA

The wall thickness, diameters, peak flow rate, resistance index (RI), proportions of luminal stenosis and luminal occlusion, and vascularization grade of the carotid artery were evaluated for their relationship with disease activity biomarkers. The results suggested that ESR, CRP, and SAA levels had a moderate relationship with wall thickness (*r* = 0.344, 0.261, and 0.392, respectively; *p* < 0.05; Fig. 2). However, the other CEUS parameters were not found to be associated with biomarkers of disease activity.

### Risk of disease activity in different vascularization grades

To further clarify the risk of disease activity in different vascularization grades, we conducted logistic regression analysis. The OR of vascularization grade 2 was 4.72 (95% CI, 1.86–11.98; *p* = 0.001) by reference to grade 0. After adjusting for ESR and CRP, the OR was still significant (OR, 3.46; 95% CI, 1.24–9.62; *p* = 0.017). The OR of developing disease activity was 4.40 (95% CI, 1.67–11.55; *p* = 0.003) when the wall thickness increased every 1 cm. After adjusting for ESR and CRP, the OR was 3.32 (95% CI, 1.15–9.62).

### Potential of carotid CEUS to identify active and inactive TA patients

ROC analyses were conducted to assess the value of carotid CEUS in distinguishing the active condition (Table 3). As for the solo ultrasound index (carotid artery wall thickness and carotid wall vascularization grade) of carotid in assessing TA activity, the sensitivity ranged from 62.5 to 78.9% and specificity ranged from 60.7 to 73.3%. An integration of the carotid artery wall thickness and vascularization grade through a parallel test resulted in an increase in sensitivity to 86.9% with area under the curve (AUC) at 0.784. For better predicting disease activity, we then conducted a serial test using the above combined CEUS parameters and ESR, the results demonstrated a sensitivity of 81.1% and specificity of 81.5% and the positive likelihood ratio and negative likelihood ratio were 4.38 and 0.23 respectively with AUC of 0.848.

**Table 3:** The value of carotid ultrasound parameters for identifying disease activity

	Optimal cut-point	AUC	95%CI	Sensitivity (%)	Specificity (%)	Positive likelihood ratio	Negative likelihood ratio
ESR, mm/H	20	0.782	(0.672,0.893)	71.8	62.1	1.89	0.45
CRP, mg/L	10	0.736	(0.620,0.852)	60.0	79.0	2.90	0.50
Carotid artery wall thickness, mm	1.75	0.725	(0.602,0.848)	78.9	60.7	2.01	0.35
Carotid wall vascularization grade	Grade 2	0.710	(0.586,0.834)	62.5	73.3	2.34	0.51
Combined CEUS parameters (parallel test)	–	0.784	(0.672,0.895)	86.8	60.7	2.21	0.22
CEUS combined ESR (serial test)	–	0.848	(0.751,0.944)	81.1	81.5	4.38	0.23
CEUS combined CRP (serial test)	–	0.821	(0.720,0.921)	73.7	74.1	2.84	0.36

*ESR* erythrocyte sedimentation rate, *CRP* C-reactive protein, *CEUS* contrast-enhanced ultrasonography

### Carotid CEUS findings during the 3-month follow-up

Thirty-eight patients completed their 3-month follow-up. Of the other 46 patients, 2 died of lung infection, 4 were lost to follow-up, and 39 did not reach the time to follow-up. Among completely followed patients, 22 had already received treatment at baseline, whereas 16 were naïve; the average age (32.18 ± 9.77 vs. 29.00 ± 9.69 years, *p* > 0.05) and disease duration (49.77 ± 36.51 vs 29.19 ± 79.90 months, *p* > 0.05) of the two groups were not significantly different. After 3 months of treatment, the proportion of disease activity, according to PGA, decreased from 65.8 to 18.4%. The ESR and CRP levels decreased from 37.84 ± 34.81 to 21.89 ± 20.26 mm/H (*p* = 0.019) and 31.11 ± 49.52 to 15.37 ± 26.25 mg/L, respectively (Table 4).

**Table 4:** Changes of inflammatory parameters during 3-month follow-up

	Baseline	3-month follow-up	*p*
*N*	38	38	
Active patients (%)	25 (65.79)	7 (18.42)	< 0.001
ESR, mm/H	37.84 ± 34.81	21.89 ± 20.26	0.019
CRP, mg/L	31.11 ± 49.52	15.37 ± 26.25	0.090
SAA, mg/L	109.26 ± 196.68	66.98 ± 139.55	0.395
Platelet count, ×10^9^/L	300.76 ± 101.82	277.11 ± 96.04	0.308
Kerr scores	2.00 ± 1.14	0.55 ± 0.76	< 0.001
ITAS 2010 scores	2.45 ± 3.77	0.87 ± 1.61	0.020

*ESR* erythrocyte sedimentation rate, *CRP* C-reactive protein, *SAA* serum amyloid A

At the end of 3 months’ follow-up, the thickness of the lesion wall was significantly improved compared to baseline (1.85 ± 0.61 mm vs 2.24 ± 0.89 mm, *p* = 0.041). The proportion of severe vascularization (grade 2) decreased from 52.6 to 34.3% (*p* = 0.036). The lumen diameter increased, though the improvement was not statistically significant. The proportion of stenosis and occlusion did not change significantly after 3 months’ treatment, suggesting that the two parameters would be changed in the chronic phase of vascular disease. In addition, peak flow rate and RI did not change significantly during follow-up.

**Fig. 3:**
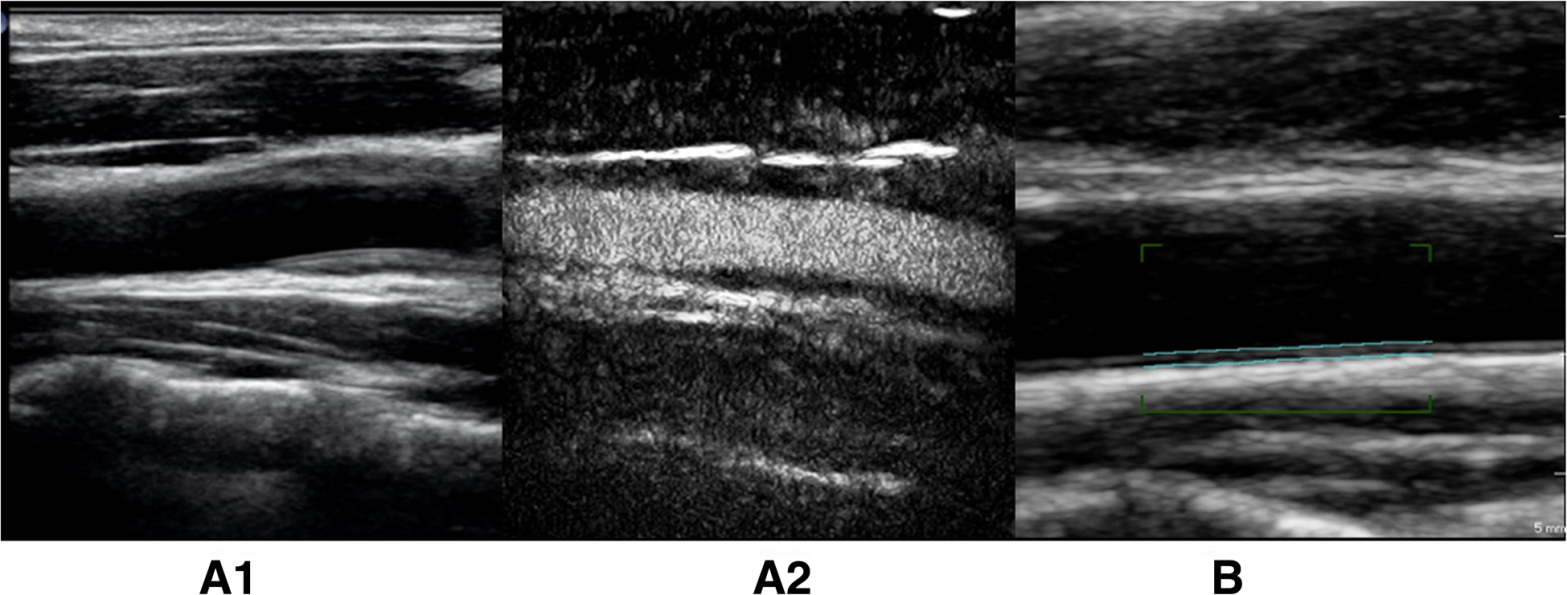
Changes of carotid CEUS before and after 3-month treatment in one TA patient. One 37-year-old male patient, who complained of dizziness for 3 months, was diagnosed with TA in our hospital. The carotid artery CDUS at baseline showed significant thickened vessel wall (**A1**). Further CEUS examination showed severe vascularization in thickened wall (**A2**). **B** After treatment of glucocorticoid and cyclophosphamide for 3 months, the original lesion had completely restored to normal

The carotid artery lesions of two patients returned completely to normal (Fig. 3) and those of another patient decreased from 2.2 to 1.1 mm. Clinical symptoms, such as fever and neck pain, in the above three patients were all relieved from baseline. The ESR level decreased from 66.00 ± 57.42 to 4.00 ± 3.46 mm/H. Among the remaining 35 patients, the ultrasound evaluation had progressed in 3 cases, improved in 10 cases, and remained unchanged in 22 cases. ESR decreased significantly in the improvement group compared with the unchanged group (9.77 ± 10.58 vs. 29.24 ± 21.84 mm/H), and there was no statistical difference in CRP. In the progression group, two patients complained of neck pain, though we observed decreases in the levels of ESR (23.00 ± 20.51 mm/H vs 59.00 ± 45.07 mm/H) and CRP (28.20 ± 47.28 mg/L vs 71.46 ± 112.02 mg/L). The results indicated that though ESR declined, the lesions of the vascular wall might still exist or even progress.

## Discussion

The biggest challenge faced by clinicians is the lack of quantitative, reliable, and effective measures to monitor disease activity in TA. From the present study, a few points can be yielded: (1) In contrast to carotid plaque, TA has the macaroni sign, which is characterized by a uniform annular thickening of the transverse section of the affected arterial wall. (2) In this study, we enrolled patients with TA alone. Compared with previous studies, our sample size was the largest. (3) The thickness and severe vascularization of the vascular wall were specific to active TA patients. These two indices were reduced after 3 months of treatment. (4) During the follow-up period, the ESR decreased, but a small amount of neovascularization signal was still visible in the vascular wall, suggesting that CEUS was more sensitive for detecting vascular inflammation.

With advancements in imaging technology, the sensitivity of detecting blood vessel inflammation and vascular lesions has been increased. Previous studies reported that common vascular involvement in Caucasian patients primarily encompasses the abdominal aorta, superior mesenteric artery, subclavian artery, and common carotid artery. In our previous TA cohort study, it was found that the left subclavian artery, left common carotid artery, brachiocephalic artery, thoracic aorta, abdominal aorta, and renal artery were commonly involved in the Chinese population, which suggested the existence of an ethnic difference. Our previous study found that type V, followed by type IIb and type I, was common among TA patients. In the present study, we found 44% of patients with types V and I, followed by 9.5% with type IIa and 2.4% with type IIb. These studies suggest that carotid artery involvement is common in patients with TA in the Chinese population. MRA is not optimal for carotid artery assessment, but ultrasound has a higher resolution both in the diagnosis and assessment of inflammatory activity in TA. Thus, ultrasound is worth being promoted or should be preferred for application in patients with carotid artery involvement.

In TA patients, although systemic inflammation is significantly associated with local inflammation of the vessel wall, they are also independent in some cases [14]. Previous studies have shown that 30 to 40% of active TA patients have normal ESR and CRP levels. Hence, the assessment of systemic inflammation alone may yield misleading results. At present, an increasing number of techniques are being used to evaluate TA activity. In fact, digital subtraction angiography has been replaced by other imaging techniques. In particular, CTA clearly demonstrates luminal stenosis, expansion, and wall calcification, which contribute to vascular involvement. In addition, MRA, CDUS, and PET/CT can be used to evaluate wall thickness and edema. However, MRA requires a long duration for examination and is associated with contrast agent toxicity, whereas PET/CT is associated with high costs and the risk of nuclear radiation.

Carotid CEUS is an effective diagnostic method for assessing the risk of cardiovascular disease. Previous studies have indicated that, on carotid CEUS, plaque neovascularization is positively correlated with inflammation activity [15]. Thus, CEUS can help assess the stability of carotid plaques by demonstrating the proliferation of neovascularization and adventitial blood vessels in the plaque, which further aids in stratifying the risk of disease. In vascular inflammatory disease, the semi-quantitative analysis of neovascularization can objectively reflect the activity of vascular inflammation. Several case reports [16, 17] found that carotid CEUS can assess wall neovascularization via a semi-quantitative technique, which clearly shows the thickness of the vascular wall and the lumen boundary. Therefore, we can use this observable technique of microvascular neovascularization in the thickened wall to evaluate TA activity.

In the present study, there were differences between the active and inactive groups in terms of the CDUS and CEUS parameters. The wall thickness of the carotid lesion was significantly greater in the active group. Carotid wall vascularization grade 2 was observed more often in the active cases. After regular treatment, these two indicators can be significantly improved, and some patients return to completely normal carotid. However, the proportions of vascular stenosis and occlusion were significantly higher in the inactive group. Notably, Fan et al. [18] reported thicker walls and a higher proportion of stenosis in active TA patients, which is inconsistent with our results. This may be due to the following reasons. First, different referential standards were used in two studies, which may lead to differences in the evaluation of active patients. Second, this research included more new-onset patients. Vascular stenosis and occlusion in TA may be a manifestation of chronic disease rather than active disease.

ROC analysis showed that the combination of CEUS parameters and ESR yielded better results than separate indicators. The optimal cutoff points for ESR, carotid artery wall thickness, and vascularization grade were 20 mm/H, 1.75 mm, and vascularization grade 2, respectively. The combined indicators could differentiate between active and inactive TA patients, with a sensitivity of 81.1% and specificity of 81.5%. Giuseppe et al. observed a significant relationship between carotid CEUS vascularization grade and PET/CT uptake. Moreover, CEUS had a sensitivity of 100% and specificity of 92% for identifying the active disease of large-vessel vasculitis using PET/CT as a reference standard, which was much higher than our findings [19]. However, the patients in their study included 17 with giant cell arteritis.

Carotid ultrasound examination can reflect vascular structural and morphological changes under the route of disease development, which help to assess the patient’s outcomes after treatment. We found the change in carotid wall thickness was the most sensitive parameter. At 3 months’ follow-up, it was observed that the thickness of the lesion wall was significantly improved than the baseline (1.85 ± 0.61 vs 2.24 ± 0.89 mm, *p* = 0.041). Makita et al. [20] observed a difference in the remission and recurrence of carotid wall thickening in a 15-year-old patient by carotid ultrasonography during a 1-year follow-up. Seth et al. [21] also confirmed that in patients with normal carotid arteries, IMT thickening was still associated with disease activity. Its sensitivity to diagnose disease activity is 82%. Furthermore, our study found that patients with decreased ESR and CRP still had a progression of vascular wall inflammation at 3 months of follow-up. The inflammation of the carotid wall in TA patients is not completely consistent with laboratory inflammation indicators.

Thus, CEUS appears to have some potential for assessing disease activity in TA and during follow-up.

The present study has certain limitations. First, the 84 cases included in this study contained newly treated patients as well as those undergoing long-term treatment. The average treatment duration was 47.49 ± 66.03 months. In our previous study, we found that carotid ultrasound contrast microbubble development was affected by artery wall fibrosis and collateral circulation in some patients, which may have overestimated contrast activity at the artery wall. Second, CEUS used a semi-quantitative assessment in the present study; hence, it may have underestimated wall inflammation.

## Conclusion

In conclusion, CEUS is a convenient and rapid noninvasive technique for assessing the inflammation of the TA carotid artery wall. CEUS can also analyze the neovascularization of the TA carotid artery wall. The moderate relationship between the grade of neovascularization in CEUS and the levels of inflammatory markers suggests CEUS can be used as an effective method to assess disease activity in patients with TA. The sensitivity and specificity of combined CEUS parameters and ESR were 81.1% and 81.5%, respectively, for predicting TA activity. Ultrasound can more sensitively reflect changes in blood vessels and inflammation compared with acute phase reactants.

## Abbreviations

AUC: Area under the curve
CDUS: Color Doppler ultrasonography
CEUS: Contrast-enhanced ultrasonography
CIs: Confidence intervals
CRP: C-reactive protein
CTA: Computed tomography angiography
DICOM: Digital imaging and communication in medicine
ESR: Erythrocyte sedimentation rate
IMT: Intima-media thickness
ITAS2010: Indian Takayasu Clinical Activity Score
MRA: Magnetic resonance angiography
ORs: Odds ratios
PET/CT: Positron emission tomography/computed tomography
PGA: Physician’s global assessment
RI: Resistance index
ROC: Receiver operating characteristic
SAA: Serum amyloid A
TA: Takayasu arteritis
